# Evaluating the Safety of *Bacillus cereus* GW-01 Obtained from Sheep Rumen Chyme

**DOI:** 10.3390/microorganisms12071457

**Published:** 2024-07-18

**Authors:** Bowen Xu, Xinyi Huang, Haixiong Qin, Ying Lei, Sijia Zhao, Shan Liu, Gang Liu, Jiayuan Zhao

**Affiliations:** 1 Key Laboratory of Land Resources Evaluation and Monitoring in Southwest, Ministry of Education, Sichuan Normal University, Chengdu 610101, China; bowenxu990302@163.com (B.X.); huangxinyi20010813@163.com (X.H.); zjyketizu602@gmail.com (H.Q.); 17208265568@163.com (Y.L.); msbyfxx@163.com (S.Z.); liushan66332023@163.com (S.L.); rh682@sohu.com (G.L.); 2 College of Life Science, Sichuan Normal University, Chengdu 610101, China

**Keywords:** *Bacillus cereus*, pathogenicity, comparative genomics, transcriptome

## Abstract

*Bacillus cereus* is responsible for 1.4–12% food poisoning outbreaks worldwide. The safety concerns associated with the applications of *B. cereus* in health and medicine have been controversial due to its dual role as a pathogen for foodborne diseases and a probiotic in humans and animals. In this study, the pathogenicity of *B. cereus* GW-01 was assessed by comparative genomic, and transcriptome analysis. Phylogenetic analysis based on a single-copy gene showed clustering of the strain GW-01, and 54 *B. cereus* strains from the NCBI were classified into six major groups (I–VI), which were then associated with the source region and sequence types (STs). Transcriptome results indicated that the expression of most genes related with toxins secretion in GW-01 was downregulated compared to that in the lag phase. Overall, these findings suggest that GW-01 is not directly associated with pathogenic *Bacillus cereus* and highlight an insightful strategy for assessing the safety of novel *B. cereus* strains.

## 1. Introduction

*Bacillus cereus* is a ubiquitous Gram-positive, facultative anaerobic, spore-forming, rod-shaped bacterium [1,2], which has been isolated from various sources, such as soil, water, sewage sludge, air, human skin, food, and vegetation [3]. The safety concerns associated with *B. cereus* have been controversial, as the bacterium acts as both a pathogen for foodborne diseases [4] and probiotic in humans and animals [5,6,7].

*B. cereus* is responsible for 1.4–12% food poisoning outbreaks worldwide [8]. In China, 13.4% of the 1082 bacterial foodborne outbreaks between 1994 and 2005 have been attributed to this pathogen [9]. Viable bacteria in the human intestine could cause diarrhea [10,11] due to cytotoxic activities associated with their primary virulent factors, including hemolysin BL (HBL) [12,13], non-hemolytic enterotoxin (nhe) [14,15], and single-protein cytotoxin (cytK) [16,17]. The emetic form of food poisoning is mainly characterized by nausea and vomiting; a minimum of 10^3^–10^5^ colony-forming unit per gram (CFU/g) bacteria in food can typically produce disease-provoking concentrations of the emetic toxin cereulide [18,19,20,21,22]. Meanwhile, *B. cereus* is also used as a probiotic by humans and for livestock [23,24,25,26,27,28]. Scharek, Altherr, Tölke and Schmidt [5] have reported that the feed supplementation of *B. cereus* var. toyoi enhanced immunity in the intestines of sows and piglets. However, almost all *B. cereus* strains contain toxin genes, with approximately 85–100% demonstrating nheABC, 40–70% exhibiting hblCDA and cytK-2 each, while negligible a percentage of stains are ces+, and typically no strain is cytK-1+. Thus, it is quite challenging to accept and understand the rationale behind the consumption of *B. cereus* as a probiotic.

Generally, the pathogenicity of *B. cereus* has been evaluated through toxicity assessments [28,29,30,31,32], environmental conditions [33], and evolutionary aspects [34]. Dietrich, Jessberger, Ehling-Schulz, Märtlbauer and Granum [4] suggest that the presence of enterotoxin genes or a certain toxin gene profile does not necessarily confirm that a *B. cereus* strain exhibits toxic activity. Cui, Märtlbauer, Dietrich, Luo, Ding and Zhu [28] suggest that the probiotic strains of *B. cereus* spp. do not produce active toxins due to the toxicity-associated genes being transcriptionally silent. Thus, it is necessary to validate the identification techniques described above.

*B. cereus* GW-01 was isolated from sheep rumen chyme [35]. The results of immune factors, organ indices, and histological observations suggested that it was not toxic to mice and could increase the body weight gain by regulating the composition of gut microbiota [36]. In this study, possible reasons for this were studied by comparative genomic analysis, transcriptomics at different growth phases. Thus, through this study, we aimed to deliver a strategy for the safety assessment of novel *B. cereus* strains.

## 2. Materials and Methods

### 2.1. Obtaining Information on Genomes

*B. cereus* GW-01 was isolated from the rumen chyme of a 5-year-old healthy female Small Tail Han sheep (102.9532° E, 37.8587° N, Wuwei City, China), which has been identified by the morphological observation, physiological and biochemical tests, 16s RNA and whose whole-genome sequence [35]. *B. cereus* ATCC 14579 is conserved in the Department of Microbiology, Sichuan Normal University. Oral drug bacterium was isolated from a brand of live *Bacillus cereus* capsules. The complete sequence information of 54 *B. cereus* strains and ATCC 14579, available as of 20 July 2021, was retrieved from the NCBI (https://www.ncbi.nlm.nih.gov/) database (Table S1).

### 2.2. Prediction of Prophages, T3SS Effector Proteins, Virulence Factors, Antimicrobial Resistance Genes, and CRISPR Candidates

Prophage was predicated by PhiSpy (https://sourceforge.net/projects/phispy/ accessed on 7 July 2024) [37], and genomic islands (GIs) were analyzed by IslandPath-DIOMB (IslandPath-DIMOB v1.0.0) [38], SIGI-HMM (https://www.pathogenomics.sfu.ca/islandviewer/upload/ accessed on 7 July 2024) [39], IslandPicker (https://www.pathogenomics.sfu.ca/islandpick_GI_datasets/ accessed on 7 July 2024) [40], and IslandViewer (Islandviewer 4—Genomic Island Prediction and Genome Visualization Tool (sfu.ca)) [41]. The type III secretion system (T3SS) effector proteins, virulence factors, and bacterial antimicrobial resistance genes were predicated by TTSS_GUI (GitHub—ttuleyb/TortoiseTTS-GUI: GradioUI for TortoiseTTS voice generation, https://github.com/ttuleyb/TortoiseTTS-GUI accessed on 7 July 2024), Virulence Factors of Pathogenic Bacteria Database (VFDB), and Comprehensive Antibiotic Resistance Database (CARD), respectively. CRISPR candidates were investigated by CRISPRfinder (https://crisprcas.i2bc.paris-saclay.fr/CrisprCasFinder/Index accessed on 7 July 2024) [42].

### 2.3. Phylogenetic and Pan Genome Analysis

The phylogenetic analysis was conducted based on the single-copy core genes of 55 *B. cereus* strains. The alignment of all orthologous genes was concatenated using TreeBeST (treebest-1.9.2_i386-win32), and the phylogenetic tree was constructed using PhyML (PhyML 3.0) [43]. Genomes were pre-processed and annotated using Prokka (Prokka v1.13.4) [44], core genes and accessory genes were built using Roary (Roary 3.7.0) [45]. The phylogenetic tree with maximum likelihood was generated based on core genes.

### 2.4. RNA Sequencing and Transcriptomics Analysis

GW-01 was inoculated in LB medium at 30 °C and 150 rpm, after which cells at lag, logarithmic, and stationary phases were harvested via centrifugation (12,000× *g*, 10 min, 4 °C) for RNA extraction. As previously reported [35], the RNA extraction and sequencing transcriptomics were performed by Shanghai Personal Biotechnology Co., Ltd. (Shanghai, China). In this process, RNA was extracted and purified using the Trizol Reagent (Invitrogen, Carlsbad, CA, USA) and the RNeasy mini kit (Qiagen, Venlo, The Netherlands). The purity and integrity were assessed using the NanoPhotometer^®^ spectrophotometer (IMPLEN, Westlake Village, CA, USA) and RNA Nano 6000 Assay Kit of the Bioanalyzer 2100 system (Agilent Technologies, Santa Clara, CA, USA), respectively. Bacterial ribosomal RNA (rRNA) was depleted using the ribo-Zero rRNA removal kit (Illumina, San Diego, CA, USA), and RNA library was prepared with Illumina TruSeq Stranded total RNA kit. Further, paired end sequencing was performed on Illumina HiSeq X Ten (China Shanghai personalbio Co., Ltd., Shanghai, China), and transcriptomic data were aligned and mapped onto the complete genome of the GW-01 [35] strain using bowtie2 (https://bowtie-bio.sourceforge.net/index.shtml accessed on 7 July 2024). Differential gene expression analysis was normalized to reads per kilobases per million reads (RPKM) values, and RNA-Seq output data were analyzed for statistical significance using the Rockhopper software (http://cs.wellesley.edu/~btjaden/Rockhopper accessed on 7 July 2024).

### 2.5. Data Analyses

The data from toxicity assessment assay were analyzed by using the SPSS 16.0 software (SPSS, Chicago, IL, USA), presented as the mean ± standard deviation (SD). One-way ANOVA was carried out to detect the statistical significance. Statistical significance was set at *p* < 0.05. The sequence alignment was performed employing Mega-X (MEGA 1.0).

## 3. Results

### 3.1. Phylogenetic Analysis of Bacillus cereus GW-01

The reputation of *B. cereus* as a significant foodborne disease pathogen has resulted in the extensive study of the population structure and phylogenetic relationships of the isolates within the group have using diverse typing methods, to help monitor the evolution of strains or to identify clones responsible for disease or food poisoning outbreaks [46,47,48,49,50,51]. Thus, it is necessary to study the safety of GW-01 through phylogenetic analysis. Interestingly, phylogenetic analysis showed that it was difficult to distinguish the *Bacillus cereus* from *Bacillus thuringiensis*, *Bacillus anthracis*, *Bacillus weihenstephanensis*, *Bacillus mycoides*, *Bacillus pseudomycoides*, *Bacillus gaemokensis*, *Bacillus manliponensis*, *Bacillus cytotoxicus*, *Bacillus toyonensis*, *Bacillus bingmayongensis*, and *Bacillus wiedmanni* [52], and most research groups have proposed them as *Bacillus cereus* sensu lato (s. l.). The pathogenicity in this group of bacteria is very diverse, thus making it challenging to distinguish bacterial species that cause disease or food poisoning outbreaks [33,53]. In this study, the Average Nucleotide Identity (ANI) value between strain GW-01 and ATCC 14579 was 91.64%, and the ANI value of these 55 strains ranged from 90% to 98% (Supplemental Document S1). Based on computational biology, some of these 55 strains did not belong to same species although all of them were classified under *B. cereus* in the NCBI by classical microbial taxonomy. Several research groups have proposed the classification by computational biology based on whole-genome sequences of *Bacillus cereus* sensu lato (s. l.). [46,54]. However, most food scholars still identified the strain by classical microbial taxonomy, which was the main reason for choosing *Bacillus cereus* species with complete sequence information from the NCBI for further analysis.

**Figure 1:**
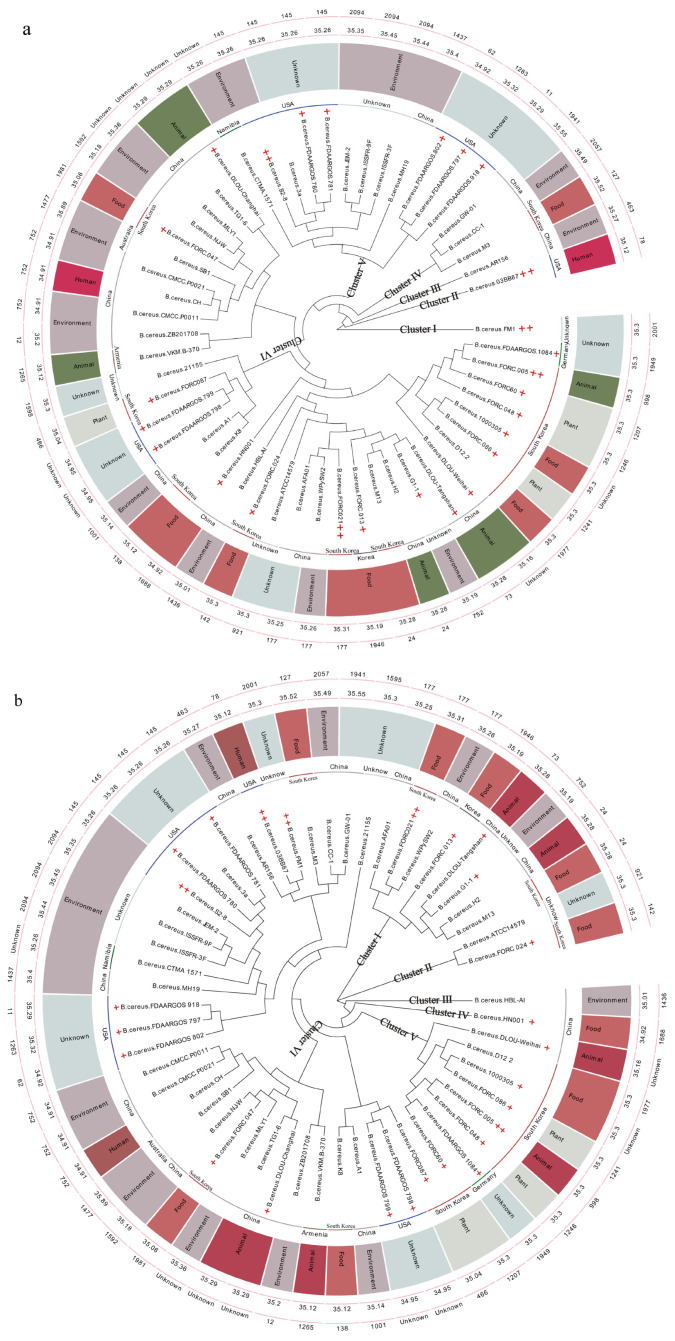
Multiple loci sequence typing (MLST, 1st layer), GC content (%, 2nd layer), habitats and hosts (3rd layer), isolating locations (4th layer), and phylogenetic analysis (5th layer) based on the single-copy core genes (**a**) and the core genes (**b**). +, and + + indicate pathogenic bacteria classified by the NCBI, and those reported by in reference.

In this study, the GC content (%) and genome size of the 55 *B. cereus* strains ranged from 34.89% to 35.55%, and 5.16 to 5.56 Mb, respectively (Figure 1a,b and Table S2). GW-01 has the highest GC content (35.55%), and its genome size is 5.24 Mb, suggesting that its evolution was different from that of other strains. Its genome size makes it difficult to analyze its pathogenicity, due to the notion that well-adapted pathogenic bacteria generally contain smaller genomes (due to less variable selection pressure) than environmental isolates [55,56]. However, this notion is debatable. Moreover, bacterial pathogenicity is associated with the habitats and hosts [57], and there is no apparent relationship between the evolution, habitats, and hosts of these 55 strains (Figure 1a,b).

Various studies have presented phylogenetic analysis based on the single-housekeeping gene [46], amplified fragment length polymorphism (AFLP) data [58,59], multi-locus sequence typing (MLST) of housekeeping genes [55,59,60,61], single-copy protein-coding genes [62], locally collinear blocks (LCBs) [63], conserved protein-coding genes [63], whole-genome single-nucleotide polymorphisms (SNPs) [55], and digital DNA-DNA hybridization (dDDH) data [64] in *Bacillus cereus* sensu lato (s. l.) strains. MLST was similarly processed in this study (Figure 1a,b). However, it is difficult to judge pathogenicity based on phylogenetic analysis [28,53,61,65].

The pathogenicity of *B. cereus* is reportedly connected to their evolution [61,66,67,68], and multiple strains from diverse habitats and hosts show genomic variations in terms of gene content, while the essential functions for a specific species are conserved as core genes that are shared among different strains [57]. In this study, phylogenetic analysis based on the single-copy core genes and the core genes was performed to determine the relationship of GW-01 and ATCC 14579 with other 53 known strains of *B. cereus* spp. (Figure 1a,b). These analyses led to clustering of the 55 *B. cereus* strains into six major groups (I–VI). Zervas et al. [69] have also suggested that *Bacillus cereus* sensu lato (s. l.) strains could be classified into six major groups by PanC and multiple loci sequence typing (MLST) characterization, and ANI-based phylogenetic trees. Our pan-genomic analyses revealed the presence of 30,868 genes in 55 *B. cereus* strains; 2221 and 22,874 of these constituted the core and accessory genomes, respectively (Figures S1 and S2). As shown in Figure 1a, most strains in cluster II and V were isolated from USA, while cluster IV and VI strains were mainly found in China and Republic of Korea (East Asia). A phylogenetic analysis based on the core genes was performed to describe the relationship of GW-01 and ATCC 14579 with the other 53 *B. cereus* strains (Figure 1b). Cluster I–V strains were mainly found in China and Republic of Korea (East Asia). These results indicated geographical association with evolution of *B. cereus* spp. However, the phylogenetic clusters based on core genes of the 55 *B. cereus* strains were not identical to those that phylogenetically divided the strains based on single-copy genes. GW-01 clustered together with CC-1 and branched near the base of the CC-1 and M3 (Figure 1b), which is consistent with the phylogenetic analysis based on single-copy gene (Figure 1a). This suggests that GW-01 likely arose before and after CC-1 and M3, respectively. Interestingly, Torres Manno, Repizo, Magni, Dunlap and Espariz [54] suggested that M3 and CC-1 were assigned to *Bacillus pacificus* and *Bacillus paranthracis*, respectively. Different species of *Bacillus cereus* sensu lato (s. l.) locating in same cluster was also reported by other studies [53,61,70]. GW-01 was not found to cluster with the known toxic strains of *B. cereus* spp. except for FORC_021 and FORC_005. Thus, not all the bacterial species within a given genetic group exhibit the same level of pathogenicity, and the distinction between pathogenic and innocuous strains remain unclear, for the entire *B. cereus* group. The pathogenicity of GW-01, CC-1, and M3 remain uncertain, but they were not clustered together with strain 03BB87, FORC_005, FORC_021, S2-8, ATCC 14579, and FM1, which have been previously reported as pathogenic.

### 3.2. Prophage, CRISPR, T3SS Effector Protein, VFDB, CARD, and Genomic Island Predictions

During evolution, the presence of prophages is an important driving factor responsible for the expression of pathogenic properties. The bacteriophage-mediated DNA transfer can convert a non-pathogenic strain into a pathogenic variety through prophage-encoded toxins, surface alterations, or increasing resistance to human immunity [71,72,73]. Marraffini Luciano and Sontheimer Erik [74] have reported the predicted role of CRISPR in limiting phage insertions. Thus, both prophage and CRISPR play an important role in virulence evolution in bacterial pathogens. However, prophage and CRISPR system in these 55 strains were not reported as related with the bacterial pathogens in this study (Supplemental Document S2). The highest GC content of prophage (66.3%) and CRISPR (68.1%) were both appeared in GW-01, indicating their difference with other strains. Moreover, T3SS effector protein, VFDB, and CARD gene were analyzed (Supplemental Document S3), but no significant association was found with the bacterial pathogens.

Genomic islands (GIs) contribute to the evolution of pathogenic microorganisms [75]. Therefore, to understand the pathogenic attributes of the 55 *B. cereus* strains, predictions of genomics islands (Gis) were performed using IslandPath-DIOMB, SIGI-HMM, IslandPicker, and IslandViewer. Highest number of genomic islands (18) were present in *B. cereus* MLY1 and DLOU-Tangshan, and least number of genomic islands (5) were identified in *B. cereus* AR156 (Table S3 and Supplemental Document S4). Notably, in *B. cereus* GW-01, five genomic islands were identified. Moreover, the highest antibiotic resistance numbers were 7 in *B. cereus* G1-1. The highest and least pathogen–host interaction (PHI) numbers were 15 in *B. cereus* 03BB87 and DLOU-Changhai each, and 2 in *B. cereus* ISSFR-9F, respectively. In strain GW-01, gene, victors gene, antibiotic resistance, and PHI numbers were 200, 12, 2, and 8, respectively. For pathogenic bacteria, the PHI number in strain 03BB87, FORC_005, FORC_021, S2-8, and FM1 are 15, 7, 9, 8, and 8, respectively; the genomic island number in these strains were 13, 11, 14, 14, and 9, respectively. Desvaux et al. [76] suggested that the functional features of PHI played an important role in bacteria pathogenicity. The gene description is shown in Supplemental Document S1. Strain GW-01 does not exhibit some of the PHIs known to be possessed by pathogenic bacteria. It suggested that strain GW-01 pathogenicity was different with these pathogenic strains.

### 3.3. Secretion Pathway of Toxin in GW-01 Based on Transcriptome Analysis

**Figure 2:**
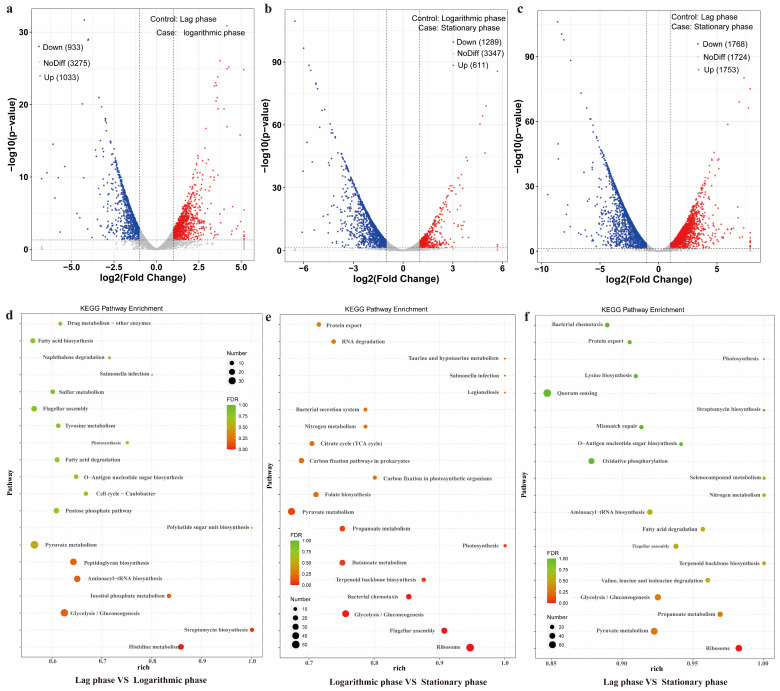
Transcriptome analysis of GW-01 at lag, logarithmic, and stationary phases. Volcano plot (**a**) and KEGG enrichment (**d**) between the lag phase and the logarithmic phase; volcano plot (**b**) and KEGG enrichment (**e**) between the lag phase and the logarithmic phase; volcano plot (**c**) and KEGG enrichment (**f**) between the lag phase and the logarithmic phase.

Although our results indicated that the evolutionary property of GW-01 was different from pathogenic *B. cereus*, its pathway of toxin secretion needed further investigation. The transcriptome results of GW-01 at lag, logarithmic, and stationary phases are shown in Figure 2. The differently expressed genes (DGEs) between lag, logarithmic, and/or stationary phases are shown in Figure 2a, Figure 2b, and Figure 2c, respectively. Compared to the lag phase, upregulated and downregulated genes were 1033 and 933 at the logarithmic phase, and were 1753 and 1768 at stationary phase, respectively. Upregulated and downregulated genes were 611 and 1289 at stationary phase compared to the logarithmic phase. The terms related with *B. cereus* growth and proliferation significantly changed as growth phase prolonged (Figure 2d–f). Interestingly, salmonella infection and flagellar assembly terms between the lag and logarithmic phases were significantly changed, suggesting that GW-01 infection might associated with its growth. Duport et al. [77] indicated that *B. cereus* exoproteome was mainly produced during the logarithmic phase.

In *Bacillus cereus* sensu lato (s. l.) strains, the diarrheal symptoms are caused by pore-forming toxins hemolysin BL (HBL), non-hemolytic enterotoxin (nhe), cytotoxin K (CytK), and hemolysin I (CLO), as well as phospholipases, proteases, and the entD protein [33]. Almost all *B. cereus* strains contain at least one type of toxic protein [4,26,78], with nhe and HBL being present in approximately 100% and 50% of all enteropathogenic *B. cereus* strains, respectively [4,79,80,81]. In this study, only nhe (A, B, and C) was found to be present in the strain GW-01. Subsequently, we attempted to analyze bacterial pathogenicity by performing the sequence alignment of toxicity-associated genes. However, it was still difficult to distinguish the pathogenicity, as the toxicity-associated genes in all the strains of *B. cereus* spp. were similar. Our analysis thus revealed that the similarity of nhe between strain GW-01 and 54 *B. cereus* strains was higher than 94% (Table S4). Toxins from certain pathogenic bacteria must cross the cell membrane(s) to gain access to their site of action in the target host cell [82]. Thus, protein secretion is important in the pathogenicity of *B. cereus* strains. Toxic protein secretion is accomplished by flagellar export apparatus (FEA), resulting its deficient secretion in non-flagellated strains [83,84]. In this study, flagellar assembly terms in logarithmic, and stationary phases were significantly downregulated compared to the lag phase. At least six integral proteins (flhA, flhB, fliO, fliP, fliQ, and fliR in Salmonella) have been reported to participate in the flagellar export [85,86], which were substantially homologous with the type III secretion system (T3SS) proteins [87,88]. In this study, T3SS analysis in 54 *B. cereus* strains revealed that the distributions of T3SS proteins between strain GW-01 and the known pathogenic strains are not regular (Table S5A). Furthermore, the amount of secreted HBL in a *B. cereus* mutant lacking flhF was shown to be significantly reduced, for which a signal recognition particle (SRP)-like GTPase is involved in the regulation of the number and arrangement of flagella on the bacterial surface [89]. The nhe secretion in *B. cereus* is influenced by T3SS proteins, flhF and flhA [90]. Similarity of genetic determinants in *B. cereus* strain GW-01 with respect to these genes in the rest of the 54 strains was higher than 90% (Table S5A). The expression of flhF, flhA, and other genes related with T3SS at the lag phase was downregulated compared to those at logarithmic, and stationary phases (Table S6), suggesting the toxins secretion in GW-01 through T3SS did not increase as growth phase prolonged.

It also has been demonstrated that HBL, nhe, and cytK secretion is dependent on the general secretory (Sec) pathway [82], yet genes encoding hbl and cytK were not detected in the strain GW-01. The proteins related with Sec path include secB, secA, srp, yidC, secYEG, ftsY, secDF-yajC, pmf, and spaces [91]. Interestingly, secB and secA were not found in GW-01, and the expression of other genes were downregulated (Table S7). These results indicated that the toxins in GW-01 might not have been secreted in Sec pathway. Huang et al. [92] have reviewed that the genetic determinants, spo0A, comER, plcR, codY, abrB, rpoN (Sigma 54), and flhA (Flagella) are associated with toxin production in *B. cereus* spp. Thus, it is necessary to investigate the similarity of these genetic determinants between strain GW-01 and 54 *B. cereus* strains in order to understand the pathogenicity of *B. cereus* strain GW-01.

**Figure 3:**
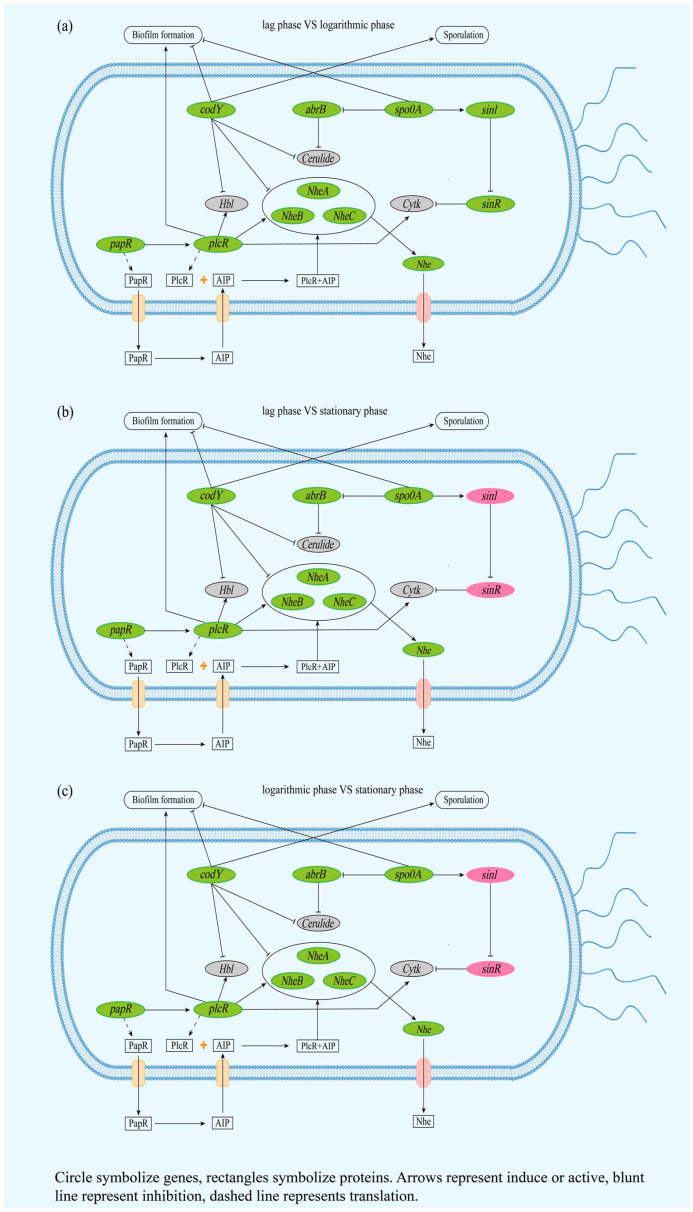
(**a**–**c**) represent the possible pathway and gene expression of toxin synthesis in lag, logarithmic, and stationary phases of GW-01. Green indicates downregulation, and red indicates upregulation.

The probable relationship between *B. cereus* toxins and regulated genes was hypothesized (Figure 3) to understand the pathogenicity of *B. cereus* strain GW-01. The spo0A protein regulating either the spore formation or biofilm development pathways in *B. cereus* [93], is responsible for the contamination of food during manufacture and processing [92]. Moreover, biofilm formation and early sporulation in *B. cereus* were also positively associated with the comER [94]. In this study, the similarity of spo0A and comER between the strain GW-01 and the rest of the 54 *B. cereus* strains was found to be higher than 97% and 94% (Table S5B), respectively, suggesting that spo0A and comER have a highly conserved amino acid sequence. Lindbäck et al. [95] have reported that codY, a global regulator, may indirectly upregulate enterotoxin production in *B. cereus*, while, flhA an important factor for toxic proteins secretion in *B. cereus*, encodes a component of flagellum-apparatus formation [92]. Our sequence alignment studies have revealed that the similarities of codY and flhA between the strain GW-01 and the rest of the 54 *B. cereus* strains were higher than 99% and 97%, respectively, indicating their highly conserved amino acid sequence (Table S5A,B). Furthermore, both the distribution and similarity of sinI and sinR in all the 53 *B. cereus* strains, as well as the strain GW-01, were irregular (Table S5A,B). These results indicated that spo0A, comER, codY and flhA are highly conserved while sinI and sinR failed to analyze the pathogenicity in the *B. cereus* strain GW-01. Hayrapetyan et al. [96] have reported that rpoN (Sigma 54) downregulates the toxin (nheA) production and sinR expression in *B. cereus* ATCC 14579. In this study, only strain GW-01 contained rpoN (Sigma 54) genes, indicating that nhe production in *B. cereus* strain GW-01 might be limited. However, plcR, which activates the expression of hbl, nhe, and cytK [92], was also present in the strain GW-01 (Figure 3). Interestingly, all the 52 *B. cereus* strains except for the strain NJ-W contain plcR genes, while the similarity between the GW-01 and NJ-W strains was found to be only 12.82%. Further, most known pathogenic *B. cereus* strains possess the papR genes; their similarities with strain GW-01 were determined to be higher than 91% (Table S5B). However, Hayrapetyan, Tempelaars, Nierop Groot and Abee [96] has reported that the expression of plcR and its regulon members was downregulated in the rpoN mutant. Thus, we speculated that the rpoN (Sigma 54) gene was associated with the toxin production either by regulating by plcR/papR or through unknown mechanisms. In this study, the expressions of genes related with toxin synthesis were downregulated except the expression of SinI and SinR at stationary phase compared with the lag phase, and the logarithmic phase, suggesting that the expression of Nhe was downregulated (Figure 3). Heilkenbrinker et al. [97] have suggested that toxicity would be reduced when the ratios of nheB and nheC are lower and higher than 50:1 and 5:1, respectively. In this study, the ratios of nheB and nheC expression at lag, logarithmic, and stationary phases were 8.7:1, 21.7:1, and 3.4:1, respectively (Table S8). Thus, we concluded that the pathogenicity of GW-01 at the lag and logarithmic phases might be higher than that at stationary phase.

## 4. Conclusions

*B. cereus* is a well-known foodborne pathogen and the toxin produced by it has been the cause of food poisoning outbreaks worldwide. However, certain strains are widely used as probiotics, which are beneficial to humans, animals and plants, as well as for the purpose of environmental restoration. Possible reasons for why GW-01 is non-pathogenic are: (1) GW-01 is derived from healthy sheep rumen chow, and its survival environment is relatively safe; (2) GW-01 was not clustered together with strain 03BB87, FORC_005, FORC_021, S2-8, ATCC 14579, and FM1, which have been previously reported as pathogenic; (3) genetic factors of toxin and pathogenic PHIs suggested that the pathogenicity of strain GW-01 was different with pathogenic strains; (4) the ratios of nheB and nheC expression were out of range for inducing toxicity.

## Data Availability

The original contributions presented in the study are included in the article and Supplementary Materials, further inquiries can be directed to the corresponding author.

## Supplementary Materials

The following supporting information can be downloaded at: https://www.mdpi.com/article/10.3390/microorganisms12071457/s1, Figure S1: Pan-genomic analyses in 55 *B. cereus* strains. Figure S2: Core and accessory genomes in 55 *B. cereus* strains. Table S1: Isolation source, geographic location and GC content of *B. cereus* used in this study. Table S2: Genome size and plasmids number in strain GW-01 and in 53 *B. cereus* strains. Table S3: Prediction of Genomics Islands (GIs) in 54 *B. cereus* strains. Table S4: Similarity of toxic genes in strain GW-01 and in 54 *B. cereus* strains. Table S5A: Similarity of genetic determinants in the *B. cereus* strain GW-01 and 53 other *B. cereus* strains. Table S5B: Similarity of genetic determinants in *B. cereus* strain GW-01 and 53 other *B. cereus* strains. Table S6: Differently expressed genes (DGEs) related with T3SS at different growth phase in *B. cereus* strain GW-01. Table S7: Differently expressed genes (DGEs) related with secretory (Sec) pathway at different growth phase in *B. cereus* strain GW-01.Table S8: Expression of nheB and nheC at lag, logarithmic, and stationary phases.
