# Supplementary material for: Evaluating the Safety of Bacillus cereus GW-01 Obtained from Sheep Rumen Chyme
Source: Microorganisms. 2024 Jul 18;12(7):1457. doi: 10.3390/microorganisms12071457 (PMC11278751; doi:10.3390/microorganisms12071457)

**Figure supplements**

Figure S1 Pan-genomic analyses in 55 *B. cereus* strains

Figure S2 Core and accessory genomes in 55 *B. cereus* strains

**Fig. S1**


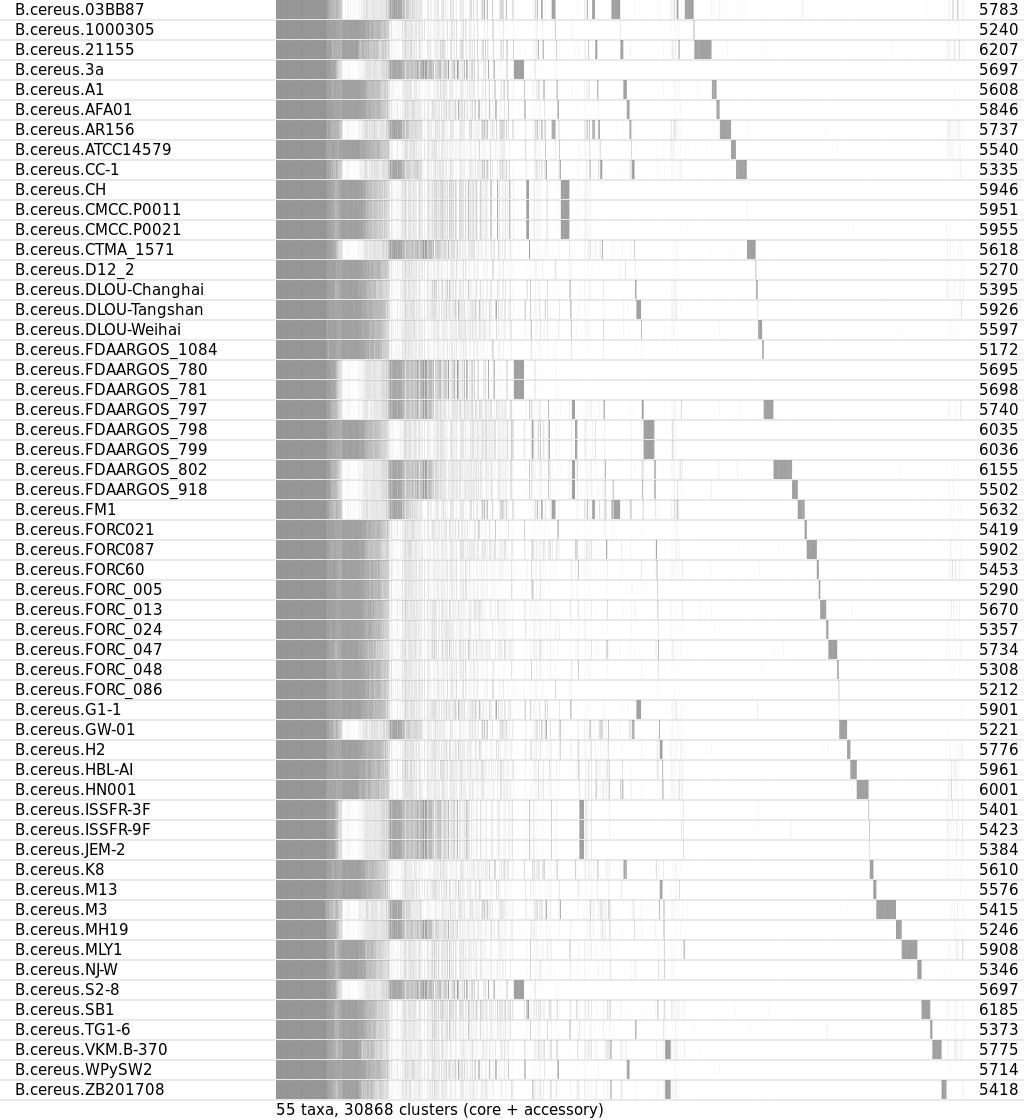


**Fig. S2**


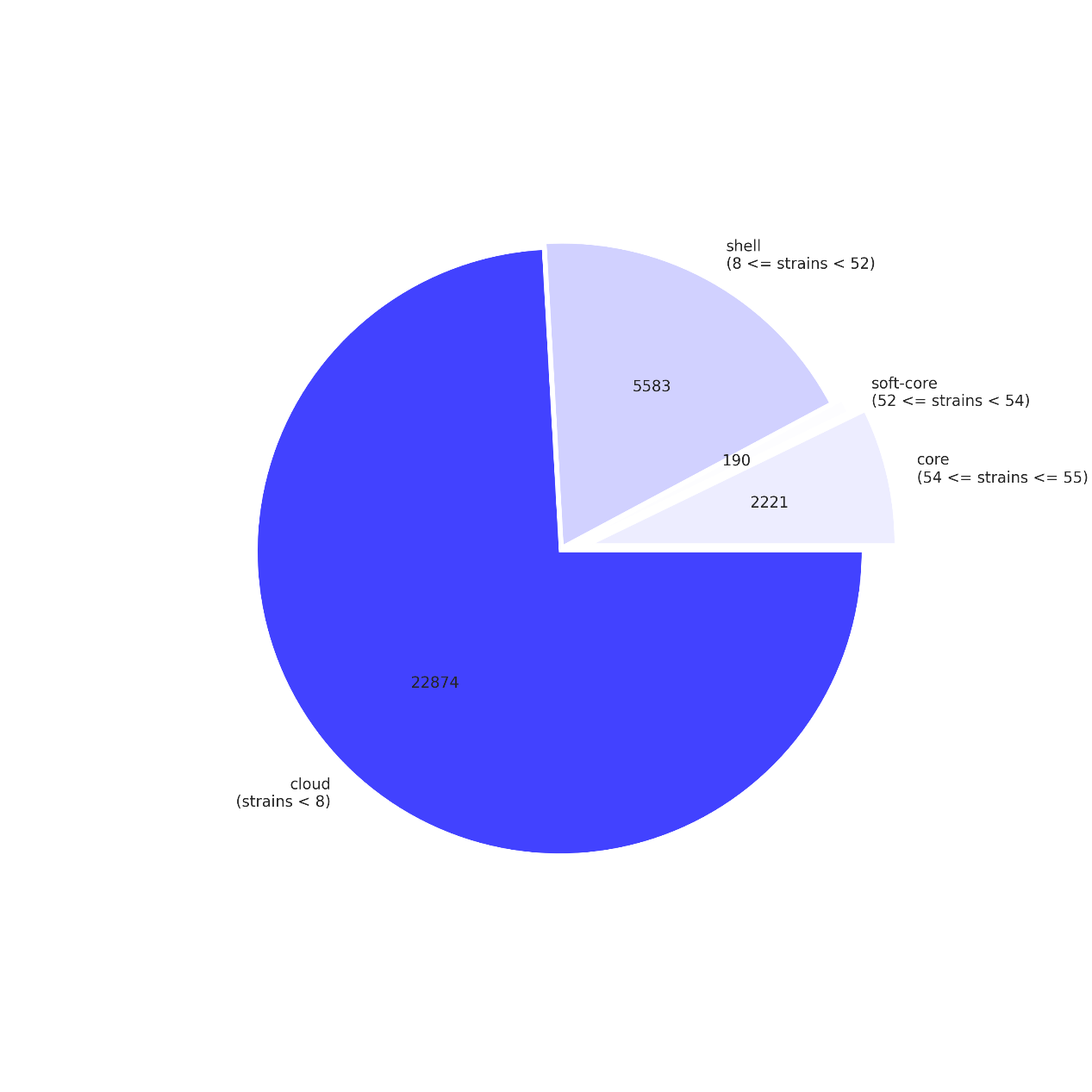

Supplement: Supplementary file 1 [file microorganisms-12-01457-s001.zip › Figure supplements.docx]
