# Supplementary material for: Evaluating the Safety of Bacillus cereus GW-01 Obtained from Sheep Rumen Chyme
Source: Microorganisms. 2024 Jul 18;12(7):1457. doi: 10.3390/microorganisms12071457 (PMC11278751; doi:10.3390/microorganisms12071457)
Supplement: Supplementary file 1 [file microorganisms-12-01457-s001.zip › Table S.doc]

Table S

**Table S1 Isolation source, geographic location and GC content of *B*. *cereus* used in this study**

| No. | Sample name | Isolation source | Geographic location | GC (%) |
| --- | --- | --- | --- | --- |
| 1 | *B*. *cereus* 03BB87 | Human | USA | 35.12 |
| 2 | *B*. *cereus* CH | Human | China | 34.91 |
| 3 | *B*. *cereus* DLOU-Changhai | Animal | China | 35.29 |
| 4 | *B*. *cereus* DLOU-Tangshan | Animal | China | 35.28 |
| 5 | *B*. *cereus* DLOU-Weihai | Animal | China | 35.16 |
| 6 | *B*. *cereus* FORC-005 | Animal | South Korea | 35.3 |
| 7 | *B*. *cereus* H2 | Animal | China | 35.28 |
| 8 | *B*. *cereus* TG1-6 | Animal | China | 35.29 |
| 9 | *B*. *cereus* VKM.B-370 | Animal | Armenia | 35.12 |
| 10 | *B*. *cereus* 1000305 | Food | South Korea | 35.3 |
| 11 | *B*. *cereus* D12-2 | Food | South Korea | 35.3 |
| 12 | *B*. *cereus* FORC021 | Food | South Korea | 35.31 |
| 13 | *B*. *cereus* FORC-013 | Food | Korea | 35.19 |
| 14 | *B*. *cereus* FORC-024 | Food | South Korea | 35.3 |
| 15 | *B*. *cereus* FORC-047 | Food | South Korea | 35.06 |
| 16 | *B*. *cereus* HN001 | Food | China | 34.92 |
| 17 | *B*. *cereus* K8 | Food | South Korea | 35.12 |
| 18 | *B*. *cereus* M13 | Food | South Korea | 35.28 |
| 19 | *B*. *cereus* M3 | Food | South Korea | 35.52 |
| 20 | *B*. *cereus* 21155 | Plant | China | 34.96 |
| 21 | *B*. *cereus* FORC-048 | Plant | South Korea | 35.3 |
| 22 | *B*. *cereus* FORC-086 | Plant | South Korea | 35.30 |
| 23 | *B*. *cereus* FORC60 | Plant | South Korea | 35.3 |
| 24 | *B*. *cereus* FORC87 | Plant | South Korea | 35.04 |
| 25 | *B*. *cereus* HBL-AI | Environment | China | 35.01 |
| 26 | *B*. *cereus* A1 | Environment | China | 35.14 |
| 27 | *B*. *cereus* AR156 | Environment | China | 35.27 |
| 28 | *B*. *cereus* CC-1 | Environment | China | 35.49 |
| 29 | *B*. *cereus* CMCC.P0011 | Environment | China | 34.91 |
| 30 | *B*. *cereus* CMCC.P0021 | Environment | China | 34.91 |
| 31 | *B*. *cereus* CTMA-1571 | Environment | Namibia | 35.26 |
| 32 | *B*. *cereus* G1-1 | Environment | Unknown | 35.19 |
| 33 | *B*. *cereus* ISSFR-3F | Environment | Unknown | 35.44 |
| 34 | *B*. *cereus* ISSFR-9F | Environment | Unknown | 35.45 |
| 35 | *B*. *cereus* JEM-2 | Environment | Unknown | 35.35 |
| 36 | *B*. *cereus* MH19 | Environment | China | 35.4 |
| 37 | *B*. *cereus* MLY1 | Environment | China | 35.36 |
| 38 | *B*. *cereus* NJ-W | Environment | China | 35.18 |
| 39 | *B*. *cereus* S2-8 | Environment | USA | 35.26 |
| 40 | *B*. *cereus* SB1 | Environment | Australia | 34.89 |
| 41 | *B*. *cereus* WPySW2 | Environment | China | 35.26 |
| 42 | *B*. *cereus* ZB201708 | Environment | China | 35.2 |
| 43 | *B*. *cereus* AFA01 | Unknown | China | 35.25 |
| 44 | *B*. *cereus* FDAARGOS-780 | Unknown | USA | 35.26 |
| 45 | *B*. *cereus* FDAARGOS-781 | Unknown | USA | 35.26 |
| 46 | *B*. *cereus* FDAARGOS-797 | Unknown | USA | 35.32 |
| 47 | *B*. *cereus* FDAARGOS-798 | Unknown | USA | 34.95 |
| 48 | *B*. *cereus* FDAARGOS-799 | Unknown | USA | 34.95 |
| 49 | *B*. *cereus* FDAARGOS-802 | Unknown | USA | 34.92 |
| 50 | *B*. *cereus* FDAARGOS-918 | Unknown | USA | 35.29 |
| 51 | *B*. *cereus* FDAARGOS-1084 | Unknown | Germany | 35.3 |
| 52 | *B*. *cereus* FM1 | Unknown | Unknown | 35.3 |
| 53 | *B*. *cereus* ATCC 14579 | Unknown | Unknown | 35.3 |
| 54 | *B*. *cereus* 3a | Unknown | USA | 35.26 |

Table S2 Genome size and plasmids number in strain GW-01 and in 53 *B*. *cereus* strains

| Number | Sample name | GC% | genome size | plasmids number |
| --- | --- | --- | --- | --- |
| 1 | Bacillus cereus 3a | 35.26 | 5.27 | 3 |
| 2 | Bacillus cereus 03BB87 | 35.12 | 5.46 | 2 |
| 3 | Bacillus cereus 21155 | 34.96 | 5.55 | 4 |
| 4 | Bacillus cereus A1 | 35.14 | 5.35 | 3 |
| 5 | Bacillus cereus AFA01 | 35.25 | 5.37 | 4 |
| 6 | Bacillus cereus 1000305 | 35.3 | 5.33 | 0 |
| 7 | Bacillus cereus AR156 | 35.27 | 5.16 | 3 |
| 8 | Bacillus cereus CC-1 | 35.49 | 5.28 | 4 |
| 9 | Bacillus cereus CH | 34.91 | 5.51 | 1 |
| 10 | Bacillus cereus CMCC.P0011 | 34.91 | 5.51 | 1 |
| 11 | Bacillus cereus CMCC.P0021 | 34.91 | 5.52 | 1 |
| 12 | Bacillus cereus CTMA-1571 | 35.26 | 5.18 | 2 |
| 13 | Bacillus cereus D12-2 | 35.3 | 5.37 | 0 |
| 14 | Bacillus cereus DLOU-Changhai | 35.29 | 5.39 | 1 |
| 15 | Bacillus cereus DLOU-Tangshan | 35.28 | 5.54 | 1 |
| 16 | Bacillus cereus DLOU-Weihai | 35.16 | 5.29 | 1 |
| 17 | Bacillus cereus FDAARGOS-780 | 35.26 | 5.27 | 3 |
| 18 | Bacillus cereus FDAARGOS-781 | 35.26 | 5.27 | 3 |
| 19 | Bacillus cereus FDAARGOS-797 | 35.32 | 5.41 | 1 |
| 20 | Bacillus cereus FDAARGOS-798 | 34.95 | 5.38 | 2 |
| 21 | Bacillus cereus FDAARGOS-799 | 34.95 | 5.38 | 2 |
| 22 | Bacillus cereus FDAARGOS-802 | 34.92 | 5.34 | 7 |
| 23 | Bacillus cereus FDAARGOS-918 | 35.29 | 5.27 | 1 |
| 24 | Bacillus cereus FDAARGOS-1084 | 35.3 | 5.32 | 0 |
| 25 | Bacillus cereus FM1 | 35.3 | 5.3 | 1 |
| 26 | Bacillus cereus FORC-005 | 35.3 | 5.35 | 0 |
| 27 | Bacillus cereus FORC-013 | 35.19 | 5.42 | 1 |
| 28 | Bacillus cereus FORC-024 | 35.3 | 5.43 | 0 |
| 29 | Bacillus cereus FORC-047 | 35.06 | 5.37 | 2 |
| 30 | Bacillus cereus FORC-048 | 35.3 | 5.36 | 0 |
| 31 | Bacillus cereus FORC-086 | 35.30 | 5.34 | 0 |
| 32 | Bacillus cereus FORC021 | 35.31 | 5.33 | 1 |
| 33 | Bacillus cereus FORC60 | 35.3 | 5.36 | 0 |
| 34 | Bacillus cereus FORC87 | 35.04 | 5.27 | 3 |
| 35 | Bacillus cereus G1-1 | 35.19 | 5.54 | 1 |
| 36 | Bacillus cereus H2 | 35.28 | 5.49 | 2 |
| 37 | Bacillus cereus HBL-AI | 35.01 | 5.48 | 1 |
| 38 | Bacillus cereus HN001 | 34.92 | 5.4 | 2 |
| 39 | Bacillus cereus ISSFR-3F | 35.44 | 5.24 | 1 |
| 40 | Bacillus cereus ISSFR-9F | 35.45 | 5.24 | 1 |
| 41 | Bacillus cereus JEM-2 | 35.35 | 5.23 | 1 |
| 42 | Bacillus cereus K8 | 35.12 | 5.33 | 2 |
| 43 | Bacillus cereus M13 | 35.28 | 5.5 | 3 |
| 44 | Bacillus cereus M3 | 35.52 | 5.22 | 1 |
| 45 | Bacillus cereus MH19 | 35.4 | 5.25 | 0 |
| 46 | Bacillus cereus MLY1 | 35.36 | 5.56 | 3 |
| 47 | Bacillus cereus NJ-W | 35.18 | 5.199 | 3 |
| 48 | Bacillus cereus S2-8 | 35.26 | 5.27 | 3 |
| 49 | Bacillus cereus SB1 | 34.89 | 5.46 | 4 |
| 50 | Bacillus cereus TG1-6 | 35.29 | 5.42 | 1 |
| 51 | Bacillus cereus VKM.B-370 | 35.12 | 5.39 | 3 |
| 52 | Bacillus cereus WPySW2 | 35.26 | 5.33 | 1 |
| 53 | Bacillus cereus ZB201708 | 35.2 | 5.47 | 0 |
| 54 | Bacillus cereus GW-01 | 35.55 | 5.24 | 0 |
| 55 | Bacillus cereus ATCC 14579 | 35.3 | 5.43 | 1 |

**Table S3** **Prediction of Genomics Islands (GIs) in 54 *B*. *cereus* strains**

| Sample | Genomic Island Number | Gene Number | Victors Gene number | Antibiotic Resistance number | PHI Number |
| --- | --- | --- | --- | --- | --- |
| *B. cereus* 03BB87 | 13 | 304 | 16 | 0 | 15 |
| *B. cereus* 1000305 | 7 | 179 | 13 | 1 | 8 |
| *B. cereus* 21155 | 15 | 336 | 5 | 3 | 5 |
| *B. cereus* 3a | 13 | 248 | 12 | 0 | 7 |
| *B. cereus* A1 | 10 | 157 | 9 | 1 | 7 |
| *B. cereus* AFA01 | 11 | 167 | 9 | 0 | 6 |
| *B. cereus* AR156 | 5 | 139 | 3 | 0 | 3 |
| *B. cereus* CC-1 | 12 | 209 | 10 | 1 | 6 |
| *B. cereus* CH | 14 | 323 | 12 | 5 | 6 |
| *B. cereus* CMCC.P0011 | 17 | 350 | 13 | 5 | 6 |
| *B. cereus* CMCC.P0021 | 14 | 318 | 12 | 5 | 6 |
| *B. cereus* CTMA_1571 | 6 | 138 | 5 | 0 | 3 |
| *B. cereus* D12_2 | 9 | 191 | 11 | 2 | 8 |
| *B. cereus* DLOU-Changhai | 13 | 345 | 0 | 5 | 15 |
| *B. cereus* DLOU-Tangshan | 18 | 296 | 14 | 5 | 8 |
| *B. cereus* DLOU-Weihai | 6 | 150 | 16 | 0 | 9 |
| *B. cereus* FDAARGOS_1084 | 10 | 203 | 11 | 0 | 9 |
| *B. cereus* FDAARGOS_780 | 15 | 283 | 13 | 0 | 8 |
| *B. cereus* FDAARGOS_781 | 12 | 225 | 7 | 0 | 5 |
| *B. cereus* FDAARGOS_797 | 15 | 215 | 10 | 0 | 5 |
| *B. cereus* FDAARGOS_798 | 17 | 337 | 17 | 3 | 13 |
| *B. cereus* FDAARGOS_799 | 14 | 274 | 11 | 2 | 9 |
| *B. cereus* FDAARGOS_802 | 15 | 251 | 13 | 4 | 7 |
| *B. cereus* FDAARGOS_918 | 7 | 135 | 9 | 0 | 4 |
| *B. cereus* FM1 | 9 | 190 | 12 | 0 | 8 |
| *B. cereus* FORC021 | 11 | 199 | 16 | 2 | 9 |
| *B. cereus* FORC087 | 17 | 322 | 14 | 2 | 12 |
| *B. cereus* FORC60 | 10 | 249 | 6 | 0 | 4 |
| *B. cereus* FORC_005 | 11 | 281 | 11 | 0 | 7 |
| *B. cereus* FORC_013 | 9 | 252 | 0 | 5 | 9 |
| *B. cereus* FORC_024 | 12 | 285 | 13 | 1 | 9 |
| *B. cereus* FORC_047 | 13 | 259 | 12 | 1 | 7 |
| *B. cereus* FORC_048 | 11 | 307 | 0 | 2 | 7 |
| *B. cereus* FORC_086 | 8 | 200 | 17 | 2 | 11 |
| *B. cereus* G1-1 | 16 | 238 | 10 | 7 | 6 |
| *B. cereus* GW-01 | 8 | 200 | 12 | 2 | 8 |
| *B. cereus* H2 | 15 | 330 | 10 | 0 | 8 |
| *B. cereus* HBL-AI | 14 | 337 | 9 | 0 | 8 |
| *B. cereus* HN001 | 11 | 197 | 0 | 0 | 4 |
| *B. cereus* ISSFR-3F | 8 | 165 | 0 | 0 | 5 |
| *B. cereus* ISSFR-9F | 6 | 134 | 3 | 0 | 2 |
| *B. cereus* JEM-2 | 8 | 166 | 3 | 0 | 5 |
| *B. cereus* K8 | 11 | 220 | 11 | 1 | 6 |
| *B. cereus* M13 | 14 | 309 | 0 | 0 | 5 |
| *B. cereus* M3 | 14 | 255 | 11 | 3 | 7 |
| *B. cereus* MH19 | 6 | 147 | 0 | 1 | 3 |
| *B. cereus* MLY1 | 18 | 345 | 15 | 1 | 9 |
| *B. cereus* NJ-W | 9 | 217 | 8 | 0 | 5 |
| *B. cereus* S2-8 | 14 | 276 | 13 | 0 | 8 |
| *B. cereus* SB1 | 16 | 273 | 10 | 3 | 5 |
| *B. cereus* TG1-6 | 12 | 285 | 0 | 4 | 9 |
| *B. cereus* VKM.B-370 | 13 | 292 | 11 | 1 | 8 |
| *B. cereus* WPySW2 | 10 | 176 | 16 | 1 | 9 |
| *B. cereus* ZB201708 | 8 | 171 | 10 | 1 | 6 |

Table S4 Similarity of toxic genes in strain GW-01 and in 54 *B*. *cereus* strains

| Sample | Gene similarity in strain GW-01 (%) | | | | | | | | | |
| --- | --- | --- | --- | --- | --- | --- | --- | --- | --- | --- |
|  | NheA | | NheB | NheC | | panC | | rpoN | Spo0A | |
| **B.cereus.03BB87** | **99.22** | **98.51** | | **94.71** |  | |  | | | **98.86** |
| B.cereus.1000305 | 96.89 | 99.01 | | 95.26 |  | |  | | | 98.11 |
| B.cereus.21155 | 96.89 | 99.01 | | 94.99 |  | |  | | | 98.11 |
| B.cereus.3a | 98.17 | 99.01 | | 94.71 | 95.04 | |  | | | 99.24 |
| B.cereus.A1 | 96.89 | 99.01 | | 95.82 |  | |  | | | 97.73 |
| B.cereus.AFA01 | 97.15 | 99.01 | | 95.54 |  | |  | | | 98.11 |
| B.cereus.AR156 | 99.22 | 98.76 | | 94.15 | 95.04 | |  | | | 98.86 |
| B.cereus.CC-1 | 97.41 | 99.50 | | - | 99.29 | |  | | | 99.62 |
| B.cereus.CH | 96.89 | 99.25 | |  |  | |  | | | 98.11 |
| B.cereus.CMCC.P0011 | 96.89 | 99.25 | |  |  | |  | | | 98.11 |
| B.cereus.CMCC.P0021 | 96.89 | 99.25 | |  |  | |  | | | 98.11 |
| B.cereus.CTMA_1571 | 98.19 | 99.01 | | 94.43 | 95.35 | |  | | | 99.24 |
| B.cereus.D12_2 | 96.63 | 99.01 | | 95.54 |  | |  | | | 98.11 |
| B.cereus.DLOU-Changhai | 96.89 | 99.50 | | 95.54 |  | |  | | | 98.11 |
| B.cereus.DLOU-Tangshan | 97.15 | 99.50 | |  |  | |  | | | 98.11 |
| B.cereus.DLOU-Weihai | 96.89 | 98.51 | | 95.54 |  | |  | | | 98.11 |
| B.cereus.FDAARGOS_1084 | 96.89 | 99.01 | | 95.54 |  | |  | | | 98.11 |
| B.cereus.FDAARGOS_780 | 98.19 | 99.01 | | 94.71 | 95.04 | |  | | | 99.24 |
| B.cereus.FDAARGOS_781 | 98.19 | 99.01 | | 94.71 | 95.04 | |  | | | 99.24 |
| B.cereus.FDAARGOS_797 | 97.41 | 99.25 | | 94.99 | 94.68 | |  | | | 99.24 |
| B.cereus.FDAARGOS_798 | 96.63 | 99.01 | | 95.54 |  | |  | | | 97.73 |
| B.cereus.FDAARGOS_799 | 96.63 | 99.01 | | 95.54 |  | |  | | | 97.73 |
| B.cereus.FDAARGOS_802 | 97.67 | 99.01 | | 94.43 |  | |  | | | 99.24 |
| B.cereus.FDAARGOS_918 | 97.67 | 99.01 | | 94.71 | 94.68 | |  | | | 99.24 |
| **B.cereus.FM1** | **99.22** | **98.51** | | **94.71** | **93.62** | |  | | | **98.86** |
| **B.cereus.FORC021** | **97.15** | **99.01** | | **95.54** |  | |  | | | **98.11** |
| B.cereus.FORC087 | 96.63 | 99.01 | | 95.26 |  | |  | | | 98.11 |
| B.cereus.FORC60 | 96.80 | 99.01 | | 95.54 |  | |  | | | 98.11 |
| **B.cereus.FORC_005** | **96.89** | **99.01** | | **94.55** |  | |  | | | **98.11** |
| B.cereus.FORC_013 | 97.15 | 99.50 | | 94.26 |  | |  | | | 98.11 |
| B.cereus.FORC_024 | 97.15 | 99.25 | | 95.54 |  | |  | | | 98.11 |
| B.cereus.FORC_047 | 97.41 | 99.50 | |  |  | |  | | | 98.11 |
| B.cereus.FORC_048 | 96.89 | 99.01 | | 95.54 |  | |  | | | 98.11 |
| B.cereus.FORC_086 | 96.89 | 99.01 | | 95.26 |  | |  | | | 98.11 |
| B.cereus.G1-1 | 97.15 | 99.50 | |  |  | |  | | | 98.11 |
| B.cereus.H2 | 97.15 | 99.50 | | 95.26 |  | |  | | | 98.11 |
| B.cereus.HBL-AI | 96.89 | 99.01 | |  |  | |  | | | 98.11 |
| B.cereus.HN001 | 96.89 | 99.01 | |  |  | |  | | | 98.11 |
| B.cereus.ISSFR-3F | 97.93 | 99.01 | | 94.43 |  | |  | | | 99.24 |
| B.cereus.ISSFR-9F | 97.93 | 99.01 | | 94.43 |  | |  | | | 99.24 |
| B.cereus.JEM-2 | 97.93 | 99.01 | | 94.43 |  | |  | | | 99.24 |
| B.cereus.K8 | 96.89 | 99.01 | | 95.82 |  | |  | | | 97.73 |
| B.cereus.M13 | 97.15 | 99.50 | | 95.26 |  | |  | | | 98.11 |
| B.cereus.M3 | 97.41 | 99.44 | | 97.21 |  | |  | | | 99.62 |
| B.cereus.MH19 | 97.67 | 98.77 | | 94.43 |  | |  | | | 99.24 |
| B.cereus.MLY1 | 97.15 | 99.50 | | 94.99 |  | |  | | | 98.11 |
| B.cereus.NJ-W | 97.41 | 99.50 | |  |  | |  | | | 98.11 |
| **B.cereus.S2-8** | **98.19** | **99.01** | | **94.71** |  | |  | | | **99.24** |
| B.cereus.SB1 | 97.15 | 99.50 | |  |  | |  | | | 98.11 |
| B.cereus.TG1-6 | 96.89 | 99.50 | |  |  | |  | | | 98.11 |
| B.cereus.VKM.B-370 | 97.15 | 99.50 | | 94.71 |  | |  | | | 97.73 |
| B.cereus.WPySW2 | 97.15 | 99.01 | | 95.54 |  | |  | | | 98.11 |
| B.cereus.ZB201708 | 97.15 | 99.50 | | 94.71 |  | |  | | | 98.11 |

**Table S5A. Similarity of genetic determinants in the *B*. *cereus* strain GW-01 and 53 other *B*. *cereus* strains**

| Strains | Similarity of genetic determinants in strain GW-01 (%) | | | | | | | | |
| --- | --- | --- | --- | --- | --- | --- | --- | --- | --- |
|  | resD | secDF | yidC | rpmF | seca | Ftsy | flhF | flhA (Flagella) |  |
| GW-01 | 100 | 100 | 100 | 100 | 100 | 100 | 100 | 100 |  |
| 03BB87 | 100 | 98.81 | 100 | 100 | 99.28 | 99.39 | 97.23 | 98.84 |  |
| 1000305 | 99.58 | 98.94 | 98.85 | 100 | 99.40 | 96.96 | 95.41 | 98.84 |  |
| 21155 | 99.58 | 98.28 | 98.85 | 100 | 98.68 | 97.26 | 94.95 | 98.84 |  |
| 3a | 100 | 99.47 | 97.69 | 100 | 99.40 | 98.48 | 95.18 | 99.27 |  |
| A1 | 99.58 | 99.34 | 95.38 | 100 | 99.04 | 96.96 | 97.94 | 98.69 |  |
| AFA01 | 99.58 | 98.67 | 99.62 | 100 | 99.16 | 97.26 | 95.87 | 98.84 |  |
| AR156 | 100 | 98.28 | 100 | 100 | 98.84 | 99.09 | 97.25 | 98.84 |  |
| CC-1 | 100 | 100 | 98.85 | 100 | 99.52 | 100 | 98.61 | 99.42 |  |
| CH | 99.58 | 97.48 | 99.62 | 100 | 99.68 | 96.96 | 94.95 | 98.40 |  |
| CMCC.P0011 | 99.58 | 97.48 | 99.62 | 100 | 99.68 | 96.96 | 94.95 | 98.26 |  |
| CMCC.P0021 | 99.58 | 97.48 | 99.62 | 100 | 99.68 | 96.96 | 94.95 | 98.40 |  |
| CTMA_1571 | 100 | 97.48 | 97.69 | 100 | 99.52 | 98.78 | 97.94 | 99.27 |  |
| D12_2 | 99.58 | 98.81 | 98.85 | 100 | 98.84 | 97.26 | 95.41 | 98.84 |  |
| DLOU-Changhai | 99.58 | 98.54 | 98.23 | 100 | 98.68 | 96.96 | 95.18 | 98.84 |  |
| DLOU-Tangshan | 99.58 | 98.67 | 99.62 | 100 | 99.16 | 97.26 | 94.95 | 98.84 |  |
| DLOU-Weihai | 99.58 | 98.67 | 98.46 | 100 | 99.04 | 97.26 | 95.87 | 98.84 |  |
| FDAARGOS_1084 | 99.58 | 98.94 | 98.46 | 100 | 99.04 | 97.26 | 95.41 | 98.69 |  |
| FDAARGOS_780 | 100 | 99.47 | 37.25 | 100 | 99.04 | 98.48 | 97.94 | 99.27 |  |
| FDAARGOS_781 | 100 | 99.47 | 97.69 | 100 | 99.04 | 98.48 | 97.94 | 99.27 |  |
| FDAARGOS_797 | 100 | 99.34 | 37.25 | 100 | 99.52 | 98.78 | 98.62 | 99.13 |  |
| FDAARGOS_798 | 99.58 | 98.54 | 95 | 100 | 99.04 | 97.26 | 94.95 | 98.84 |  |
| FDAARGOS_799 | 99.58 | 98.54 | 38.04 | 100 | 99.04 | 97.26 | 95.41 | 98.84 |  |
| FDAARGOS_802 | 99.58 | 99.34 | 37.25 | 100 | 99.04 | 99.09 | 98.17 | 98.84 |  |
| FDAARGOS_918 | 100 | 99.34 | 97.69 | 100 | 99.04 | 99.09 | 98.62 | 98.98 |  |
| FM1 | 100 | 98.81 | 100 | 100 | 99.04 | 99.39 | 98.62 | 99.13 |  |
| FORC021 | 99.58 | 98.67 | 99.62 | 100 | 99.16 | 97.26 | 94.95 | 98.84 |  |
| FORC087 | 99.58 | 98.81 | 95.38 | 100 | 99.16 | 97.26 | 95.64 | 98.69 |  |
| FORC60 | 99.58 | 98.94 | 98.85 | 100 | / | 97.26 | 95.87 | 98.84 |  |
| FORC_005 | 99.58 | 98.94 | 98.85 | 100 | 99.04 | 97.26 | 99.23 | 98.84 |  |
| FORC_013 | 99.58 | 98.81 | 99.62 | 100 | 99.04 | 97.26 | 95.41 | 98.84 |  |
| FORC_024 | 99.58 | 98.81 | 99.62 | 100 | 99.16 | 96.96 | 95.41 | 98.84 |  |
| FORC_047 | 99.58 | 98.81 | 99.62 | 100 | 99.44 | 96.96 | 95.18 | 98.40 |  |
| FORC_048 | 99.58 | 98.94 | 98.85 | 100 | 99.04 | 97.26 | 95.41 | 98.69 |  |
| FORC_086 | 99.58 | 98.94 | 98.85 | 100 | 99.04 | 97.26 | 95.41 | 98.84 |  |
| G1-1 | 99.58 | 98.67 | 99.62 | 100 | 99.16 | 97.26 | 94.95 | 98.84 |  |
| H2 | 99.58 | 98.80 | 98.85 | 100 | 99.16 | 97.26 | 94.95 | 97.83 |  |
| HBL-AI | 99.58 | 98.80 | 98.85 | 100 | 99.16 | 97.26 | 94.03 | 98.84 |  |
| HN001 | 99.58 | 98.94 | 98.85 | 100 | 99.16 | 97.26 | 94.72 | 98.69 |  |
| ISSFR-3F | 100 | 99.47 | 37.25 | 100 | 99.40 | 99.09 | 99.08 | 99.13 |  |
| ISSFR-9F | 100 | 99.47 | 97.69 | 100 | 99.40 | 99.09 | 99.08 | 99.13 |  |
| JEM-2 | 100 | 99.47 | 37.25 | 100 | 99.40 | 99.09 | 99.08 | 99.13 |  |
| K8 | 99.58 | 98.54 | 95 | 100 | 99.16 | 97.26 | 94.95 | 98.69 |  |
| M13 | 100 | 98.81 | 98.85 | 100 | 99.16 | 97.26 | 94.95 | 98.84 |  |
| M3 | 99.58 | 99.07 | 98.08 | 100 | 99.76 | 99.67 | 98.62 | / |  |
| MH19 | 100 | 99.47 | 98.08 | 100 | 99.40 | 99.09 | 97.71 | 99.42 |  |
| MLY1 | 99.58 | 98.41 | 98.11 | 100 | 98.56 | 96.96 | 94.72 | 98.40 |  |
| NJ-W | 99.58 | 97.61 | 36.47 | 100 | 98.56 | 96.96 | 94.95 | 98.55 |  |
| S2-8 | 100 | 99.47 | 97.69 | 100 | 99.40 | 98.48 | 97.94 | 99.27 |  |
| SB1 | 99.58 | 97.35 | 99.62 | 100 | 98.56 | 96.96 | 95.18 | 98.26 |  |
| TG1-6 | 99.58 | 98.67 | 36.47 | 100 | 98.68 | 96.96 | 95.18 | 98.84 |  |
| VKM.B-370 | 99.58 | 98.41 | 98.08 | 100 | 98.80 | 96.35 | 94.50 | 98.84 |  |
| WPySW2 | 99.58 | 98.67 | 37.98 | 100 | 99.16 | 97.26 | 95.87 | 98.84 |  |
| ZB201708 | 99.58 | 97.65 | 98.08 | 100 | 98.80 | 96.35 | 94.72 | 98.84 |  |

**Table S5B. Similarity of genetic determinants in *B. cereus* strain GW-01 and 53 other *B*. *cereus* strains**

| Strains | Similarity of genetic determinants in strain GW-01 (%) | | | | | | | |
| --- | --- | --- | --- | --- | --- | --- | --- | --- |
|  | Spo0A | ComER | PlcR | CodY | RpoN | PapR | Sinl | SinR |
| GW-01 | 100 | 100 | 100 | 100 | 100 | 100 | 100 | 100 |
| **03BB87** | **98.86** | **98.18** | **/** | **99.23** | **/** | **97.92** | **/** |  |
| 1000305 | 98.11 | 95.27 | / | 99.23 | / | / | 88.64 |  |
| 21155 | 98.11 | 94.55 | / | 99.23 | / | / | / |  |
| 3a | 99.24 | 97.45 | / | 100 | / | 91.67 | / |  |
| A1 | 97.73 | 94.91 | / | 99.23 | / | / | / |  |
| AFA01 | 98.11 | 95.64 | / | 99.23 | / | / | / |  |
| AR156 | 98.86 | 98.18 | / | 100 | / | 97.92 | / |  |
| CC-1 | 99.62 | 98.55 | / | 100 | / | 100 | / |  |
| CH | 98.11 | 95.64 | / | 99.23 | / | / | / |  |
| CMCC.P0011 | 98.11 | 95.64 | / | 99.23 | / | / | / |  |
| CMCC.P0021 | 98.11 | 95.64 | / | 99.23 | / | / | / |  |
| CTMA_1571 | 99.24 | 98.18 | / | 100 | / | / | / |  |
| D12_2 | 98.11 | 95.64 | / | 99.23 | / | / | / |  |
| DLOU-Changhai | 98.11 | 95.64 | / | 99.23 | / | / | / |  |
| DLOU-Tangshan | 98.11 | 95.64 | / | 99.23 | / | / | / |  |
| DLOU-Weihai | 98.11 | 95.64 | / | 99.23 | / | / | / |  |
| FDAARGOS_1084 | 98.11 | 95.64 | / | 99.23 | / | / | / |  |
| FDAARGOS_780 | 99.24 | 97.45 | / | 100 | / | 91.62 | 90.91 |  |
| FDAARGOS_781 | 99.24 | 97.45 | / | 100 | / | 91.62 | 90.91 |  |
| FDAARGOS_797 | 99.24 | 98.18 | / | 100 | / | / | 90.91 |  |
| FDAARGOS_798 | 97.73 | 94.91 | / | 99.23 | / | / | 88.64 |  |
| FDAARGOS_799 | 97.73 | 94.91 | / | 99.23 | / | / | 88.64 |  |
| FDAARGOS_802 | 99.24 | 98.18 | / | 100 | / | / | 90.91 |  |
| FDAARGOS_918 | 99.24 | 98.18 | / | 100 | / | / | / |  |
| **FM1** | **98.86** | **98.18** | **/** | **99.23** | **/** | **97.92** | **/** |  |
| **FORC021** | **98.11** | **95.64** | **/** | **99.23** | **/** | **/** | **/** |  |
| FORC087 | 98.11 | 94.91 | / | 99.23 | / | / | 88.64 |  |
| FORC60 | 98.11 | 95.64 | / | 99.23 | / | / | 88.64 |  |
| FORC_005 | 98.11 | 95.28 | / | 99.23 | / | / | / |  |
| FORC_013 | 98.11 | 95.64 | / | 99.23 | / | / | / |  |
| FORC_024 | 98.11 | 95.64 | / | 99.23 | / | / | / |  |
| FORC_047 | 98.11 | 95.64 | / | 99.23 | / | / | 88.64 |  |
| FORC_048 | 98.11 | 95.28 | / | 99.23 | / | / | 88.64 |  |
| FORC_086 | 98.11 | 95.28 | / | 99.23 | / | / | / |  |
| G1-1 | 98.11 | 95.64 | / | 99.23 | / | / | / |  |
| H2 | 98.11 | 95.64 | / | 99.23 | / | / | 88.64 |  |
| HBL-AI | 98.11 | 95.64 | / | 99.23 | / | / | / |  |
| HN001 | 98.11 | 95.64 | / | 99.23 | / | / | / |  |
| ISSFR-3F | 99.24 | 97.45 | / | 100 | / | 93.75 | / |  |
| ISSFR-9F | 99.24 | 97.45 | / | 100 | / | 93.75 | / |  |
| JEM-2 | 99.24 | 97.45 | / | 100 | / | 93.75 | / |  |
| K8 | 97.73 | 94.91 | / | 99.23 | / | / | / |  |
| M13 | 98.11 | 95.64 | / | 99.23 | / | / | / |  |
| M3 | 99.62 | 98.55 | / | 100 | / | 93.75 | / |  |
| MH19 | 99.24 | 95.64 | / | 100 | / | 93.75 | / |  |
| MLY1 | 98.11 | 95.64 | / | 99.23 | / | / | / |  |
| NJ-W | 98.11 | 95.64 | 12.82 | 99.23 | / | / | 88.64 |  |
| **S2-8** | **99.24** | **97.45** | **/** | **100** | **/** | **91.67** | **/** |  |
| SB1 | 98.11 | 95.64 | / | 99.23 | / | / | / |  |
| TG1-6 | 98.11 | 95.64 | / | 99.23 | / | / | / |  |
| VKM.B-370 | 97.73 | 95.64 | / | 99.23 | / | / | / |  |
| WPySW2 | 98.11 | 95.64 | / | 99.23 | / | / | 88.64 |  |
| ZB201708 | 98.11 | 95.64 | / | 99.23 | / | / | / |  |

Table S6 Differently expressed genes (DGEs) related with T3SS at different growth phase in *B*. *cereus* strain GW-01

| ID | Name | Growth phase | log_2_FoldChange | padj | Description |
| --- | --- | --- | --- | --- | --- |
| Gene1646 | *flhA* | KC_vs_KD | -1.118 | 0.003317 | flagellar biosynthesis protein FlhA |
|  |  | KD_vs_KW | -1.926 | 0.0002586 |  |
|  |  | KC_vs_KW | -3.025 | 1.015 |  |
| Gene1626 | *fliI* | KC_vs_KD | -1.661 | 6.948 | flagellum-specific ATP synthase |
|  |  | KD_vs_KW | -2.095 | 1.217 |  |
|  |  | KC_vs_KW | -3.738 | 1.738 |  |
| Gene1623 | *fliF* | KC_vs_KD | -1.454 | 0.00006533 | flagellar M-ring protein FliF |
|  |  | KD_vs_KW | -3.246 | 2.586 |  |
|  |  | KC_vs_KW | -4.682 | 2.017 |  |
| Gene1630 | *flgE* | KC_vs_KD | -0.08961 | 0.8361 | flagellar hook protein FlgE |
|  |  | KD_vs_KW | -1.896 | 5.752 |  |
|  |  | KC_vs_KW | -1.969 | 5.908 |  |
| Gene1644 | *fliR* | KC_vs_KD | -1.116 | 0.005388 | flagellar biosynthesis protein FliR |
|  |  | KD_vs_KW | -1.564 | 9.949 |  |
|  |  | KC_vs_KW | -2.661 | 5.621 |  |
| Gene1605 | *ytxE* | KC_vs_KD | -1.419 | 0.0001416 | flagellar motor protein MotS |
|  |  | KD_vs_KW | -2.332 | 9.874 |  |
|  |  | KC_vs_KW | -3.737 | 6.940 |  |
| Gene1604 | *ytxD* | KC_vs_KD | -0.4739 | 0.2353 | flagellar motor protein MotP |
|  |  | KD_vs_KW | -2.434 | 8.326 |  |
|  |  | KC_vs_KW | -2.892 | 9.582 |  |
| Gene4377 | *motA* | KC_vs_KD | -1.548 | 0.00002929 |  |
|  |  | KD_vs_KW | -2.600 | 4.087 | hypothetical protein bcere0001_41870 |
|  |  | KC_vs_KW | -4.134 | 4.071 |  |
| Gene1621 | *flgC* | KC_vs_KD | -1.849 | 0.000001296 | flagellar basal body rod protein FlgC |
|  |  | KD_vs_KW | -1.297 | 0.0006412 |  |
|  |  | KC_vs_KW | -3.125 | 1.662 |  |
| Gene1629 | *ylxG* | KC_vs_KD | -0.7260 | 0.05944 | flagellar basal body rod modification protein FlgD |
|  |  | KD_vs_KW | -1.779 | 5.708 |  |
|  |  | KC_vs_KW | -2.490 | 8.695 |  |
| Gene4376 | *motB* | KC_vs_KD | -1.740 | 2.352 | OmpA/MotB domain protein |
|  |  | KD_vs_KW | -3.937 | 5.271 |  |
|  |  | KC_vs_KW | -5.663 | 1.160 |  |
| Gene1624 | *fliG* | KC_vs_KD | -1.335 | 0.0003185 | flagellar motor switch protein FliG |
|  |  | KD_vs_KW | -2.551 | 7.305 |  |
|  |  | KC_vs_KW | -3.869 | 8.456 |  |
| Gene1639 | *fliM* | KC_vs_KD | -0.5871 | 0.1472 | flagellar motor switch protein FliM |
|  |  | KD_vs_KW | -1.213 | 0.00001214 |  |
|  |  | KC_vs_KW | -1.781 | 5.058 |  |
| Gene1608 | *fliY* | KC_vs_KD | -1.276 | 0.0004898 | flagellar motor switch protein FliN |
|  |  | KD_vs_KW | -1.491 | 1.439 |  |
|  |  | KC_vs_KW | -2.750 | 1.049 |  |
| Gene4153 | *sigA* | KC_vs_KD | -1.588 | 0.00001381 | RNA polymerase sigma factor RpoD |
|  |  | KD_vs_KW | -1.690 | 2.427 |  |
|  |  | KC_vs_KW | -3.265 | 5.892 |  |
| Gene1625 | *-* | KC_vs_KD | -1.843 | 3.066 | hypothetical protein bcere0001_15220 |
|  |  | KD_vs_KW | -1.270 | 0.0001089 |  |
|  |  | KC_vs_KW | -3.095 | 1.186 |  |
| Gene1621 | *flgC* | KC_vs_KD | -1.849 | 0.000001296 | flagellar basal body rod protein FlgC |
|  |  | KD_vs_KW | -1.297 | 0.0006412 |  |
|  |  | KC_vs_KW | -3.125 | 1.662 |  |
| Gene1620 | *flgB* | KC_vs_KD | -1.529 | 0.004331 | flagellar basal-body rod protein FlgB |
|  |  | KD_vs_KW | -0.9311 | 0.08089 |  |
|  |  | KC_vs_KW | -2.439 | 2.064 |  |
| Gene4377 | *motA* | KC_vs_KD | -1.548 | 0.00002929 | hypothetical protein bcere0001_41870 |
|  |  | KD_vs_KW | -2.601 | 4.087 |  |
|  |  | KC_vs_KW | -4.134 | 4.071 |  |
| Gene1617 | *fliD* | KC_vs_KD | -0.2759 | 0.5059 | flagellar cap protein FliD |
|  |  | KD_vs_KW | -4.193 | 4.258 |  |
|  |  | KC_vs_KW | -4.453 | 1.621 |  |
| Gene1642 | *fliP* | KC_vs_KD | -0.7851 | 0.05439 | flagellar biosynthesis protein flip |
|  |  | KD_vs_KW | -1.901 | 4.176 |  |
|  |  | KC_vs_KW | -2.670 | 1.272 |  |
| Gene1615 | *flgK* | KC_vs_KD | -0.4959 | 0.1831 | flagellar hook-associated protein FlgK |
|  |  | KD_vs_KW | -3.652 | 2.381 |  |
|  |  | KC_vs_KW | -4.132 | 9.753 |  |
| Gene1648 | *flgG* | KC_vs_KD | -1.903 | 4.750 | flagellar basal body rod protein FlgG |
|  |  | KD_vs_KW | -2.170 | 0.0002710 |  |
|  |  | KC_vs_KW | -4.057 | 2.079 |  |
| Gene1616 | *flgL* | KC_vs_KD | -0.2838 | 0.4833 | flagellar hook-associated protein FlgL |
|  |  | KD_vs_KW | -3.717 | 8.012 |  |
|  |  | KC_vs_KW | -3.984 | 1.155 |  |
| Gene826 | *tcyA* | KC_vs_KD | -1.842 | 7.668 | L-cystine-binding protein TcyA |
|  |  | KD_vs_KW | -1.062 | 0.0001734 |  |
|  |  | KC_vs_KW | -2.888 | 1.356 |  |
| Gene1635 | *flaA* | KC_vs_KD | 0.1556 | 0.6909 | flagellin |
|  |  | KD_vs_KW | -1.374 | 0.0001112 |  |
|  |  | KC_vs_KW | -1.204 | 0.0001937 |  |
| Gene1638 | *-* | KC_vs_KD | 0.2858 | 0.6586 | flagellar motor switch protein, putative |
|  |  | KD_vs_KW | -2.914 | 1.729 |  |
|  |  | KC_vs_KW | -2.613 | 0.00001136 |  |
| Gene1645 | *flhB* | KC_vs_KD | -1.312 | 0.0005813 | flagellar biosynthesis protein FlhB |
|  |  | KD_vs_KW | -1.139 | 0.00005568 |  |
|  |  | KC_vs_KW | -2.433 | 1.236 |  |
| Gene1636 | *fliC* | KC_vs_KD | -2.167 | 4.308 | flagellin |
|  |  | KD_vs_KW | -5.364 | 1.339 |  |
|  |  | KC_vs_KW | -7.519 | 7.345 |  |
| Gene1640 | *fliN* | KC_vs_KD | -0.9820 | 0.02398 | flagellar motor switch protein FliN |
|  |  | KD_vs_KW | -2.566 | 0.0003924 |  |
|  |  | KC_vs_KW | -3.530 | 3.857 |  |
| Gene1622 | *fliE* | KC_vs_KD | -1.259 | 0.0030973 | flagellar hook-basal body protein FliE |
|  |  | KD_vs_KW | -0.03461 | 0.9429 |  |
|  |  | KC_vs_KW | -1.284 | 0.0003482 |  |
| Gene1645 | *flhB* | KC_vs_KD | -1.312 | 0.0005813 | flagellar biosynthesis protein FlhB |
|  |  | KD_vs_KW | -1.139 | 0.00005568 |  |
|  |  | KC_vs_KW | -2.433 | 1.236 |  |
| Gene1618 | - | KC_vs_KD | -0.5389 | 0.1569 | flagellar protein FliS |
|  |  | KD_vs_KW | -4.543 | 1.187 |  |
|  |  | KC_vs_KW | -5.062 | 1.824 |  |
| Gene4962 | *sigL* | KC_vs_KD | 0.2944 | 0.4980 | RNA polymerase factor sigma-54 |
|  |  | KD_vs_KW | -1.159 | 7.880 |  |
|  |  | KC_vs_KW | -0.8515 | 0.005118 |  |
|  |  |  |  |  |  |

Note: KC indicated lag phase; KD indicated logarithmic phase; KW indicated stationary phase.

Table S7 Differently expressed genes (DGEs) related with secretory (Sec) pathway at different growth phase in *B*. *cereus* strain GW-01

| ID | Name | Growth phase | log_2_FoldChange | padj | Description |
| --- | --- | --- | --- | --- | --- |
| Gene849 | *secB* | KC_vs_KD | -2.345 | 1.095 | accessory Sec system S-layer assembly protein |
|  |  | KD_vs_KW | -2.070 | 4.279 |  |
|  |  | KC_vs_KW | -4.396 | 2.754 |  |
| Gene850 | *secA2* | KC_vs_KD | -2.217 | 5.674 | accessory Sec system translocase SecA2 |
|  |  | KD_vs_KW | -1.481 | 1.803 |  |
|  |  | KC_vs_KW | -3.680 | 3.764 |  |
| Gene5007 | *secA* | KC_vs_KD | -0.6322 | 0.09230 |  |
|  |  | KD_vs_KW | -2.572 | 1.163 | protein translocase subunit SecA 1 |
|  |  | KC_vs_KW | -3.187 | 4.055 |  |
| Gene3641 | *srp* | KC_vs_KD | 0.007444 | 0.9938 | hypothetical protein |
|  |  | KD_vs_KW | -3.115 | 2.886 |  |
|  |  | KC_vs_KW | -3.093 | 1.216 |  |
| Gene4868 | *yidC1* | KC_vs_KD | -0.9203 | 0.01426 | Membrane protein oxaA 2 |
|  |  | KD_vs_KW | -1.811 | 1.613 |  |
|  |  | KC_vs_KW | -2.717 | 6.291 |  |
| Gene5312 | *yidC2* | KC_vs_KD | -2.424 | 2.867 | OxaA precursor |
|  |  | KD_vs_KW | -1.779 | 7.762 |  |
|  |  | KC_vs_KW | -4.186 | 2.890 |  |
| Gene132 | *secY* | KC_vs_KD | -0.1408 | 0.7930 | preprotein translocase subunit SecY |
|  |  | KD_vs_KW | -3.347 | 8.592 |  |
|  |  | KC_vs_KW | -3.474 | 1.866 |  |
| Gene2577 | *secY* | KC_vs_KD | -0.2857 | 0.5217 | preprotein translocase subunit SecY |
|  |  | KD_vs_KW | -0.8304 | 0.001749 |  |
|  |  | KC_vs_KW | -1.100 | 0.0006648 |  |
| Gene97 | *secE* | KC_vs_KD | -2.426 | 9.716 |  |
|  |  | KD_vs_KW | -1.794 | 6.143 | preprotein translocase subunit SecE |
|  |  | KC_vs_KW | -4.199 | 1.137 |  |
| Gene4951 | *secG* | KC_vs_KD | -0.2132 | 0.7852 | preprotein translocase subunit SecG |
|  |  | KD_vs_KW | -0.07813 | 0.9173 |  |
|  |  | KC_vs_KW | -0.2730 | 0.6327 |  |
| Gene3642 | *ftsY* | KC_vs_KD | -0.7041 | 0.05597 | signal recognition particle-docking protein FtsY |
|  |  | KD_vs_KW | -2.163 | 5.276 |  |
|  |  | KC_vs_KW | -2.852 | 1.698 |  |
| Gene4269 | *secDF* | KC_vs_KD | -1.355 | 0.0002376 | protein translocase subunit SecDF |
|  |  | KD_vs_KW | -2.216 | 3.091 |  |
|  |  | KC_vs_KW | -3.552 | 3.987 |  |
| Gene4274 | *yrbF* | KC_vs_KD | -1.394 | 0.004009 | preprotein translocase subunit YajC |
|  |  | KD_vs_KW | -0.8453 | 0.03383 |  |
|  |  | KC_vs_KW | -2.222 | 0.00001718 |  |
|  | *pmf* | KC_vs_KD |  |  |  |
|  |  | KD_vs_KW |  |  |  |
|  |  | KC_vs_KW |  |  |  |
|  | *spases* | KC_vs_KD |  |  |  |
|  |  | KD_vs_KW |  |  |  |
|  |  | KC_vs_KW |  |  |  |
| Gene1647 | *flhF* | KC_vs_KD | -1.709 | 4.285 | flagellar biosynthesis protein FlhF |
|  |  | KD_vs_KW | -2.460 | 6.423 |  |
|  |  | KC_vs_KW | -4.153 | 5.164 |  |
| Gene1646 | *flhA* | KC_vs_KD | -1.118 | 0.003317 | flagellar biosynthesis protein FlhA |
|  |  | KD_vs_KW | -1.926 | 0.0002586 |  |
|  |  | KC_vs_KW | -3.025 | 1.015 |  |
| Gene4035 | *spo0A* | KC_vs_KD | -0.8837 | 0.01468 | sporulation transcription factor Spo0A |
|  |  | KD_vs_KW | -1.974 | 3.786 |  |
|  |  | KC_vs_KW | -2.844 | 4.904 |  |
| Gene4188 | *comER* | KC_vs_KD | 0.2656 | 0.5986 | late competence protein ComER |
|  |  | KD_vs_KW | 0.3891 | 0.1900 |  |
|  |  | KC_vs_KW | 0.6637 | 0.0577 |  |
| Gene3623 | *codY* | KC_vs_KD | -0.9371 | 0.01361 | GTP-sensing pleiotropic transcriptional regulator CodY |
|  |  | KD_vs_KW | -2.002 | 0.00002889 |  |
|  |  | KC_vs_KW | -2.919 | 8.125 |  |
| Gene5175 | *plcR* | KC_vs_KD | -1.339 | 0.0002763 | transcriptional regulator |
|  |  | KD_vs_KW | -2.201 | 4.511 |  |
|  |  | KC_vs_KW | -3.522 | 2.653 |  |
| Gene1896 | *abrB* | KC_vs_KD | -0.9227 | 0.02479 | Transition state regulatory protein |
|  |  | KD_vs_KW | -0.8436 | 0.006637 |  |
|  |  | KC_vs_KW | -1.748 | 8.146 |  |
| Gene36 | *abrB* | KC_vs_KD | -0.6537 | 0.09876 | transcriptional regulator, AbrB family |
|  |  | KD_vs_KW | -3.603 | 3.298 |  |
|  |  | KC_vs_KW | -4.249 | 1.036 |  |
| Gene4962 | *sigL* | KC_vs_KD | 0.2944 | 0.4980 | RNA polymerase factor sigma-54 |
|  |  | KD_vs_KW | -1.159 | 7.880 |  |
|  |  | KC_vs_KW | -0.8515 | 0.005118 |  |
| Gene1646 | *flhA* | KC_vs_KD | -1.118 | 0.003317 | flagellar biosynthesis protein FlhA |
|  |  | KD_vs_KW | -1.926 | 0.0002586 |  |
|  |  | KC_vs_KW | -3.025 | 1.015 |  |
| Gene1253 | *sinl* | KC_vs_KD | -1.074 | 0.4061 | DNA-binding anti-repressor SinI |
|  |  | KD_vs_KW | 3.187 | 5.029 |  |
|  |  | KC_vs_KW | 2.134 | 2.481 |  |
| Gene1252 | *sinR* | KC_vs_KD | -0.06591 | 0.8978 | transcriptional regulator, partial |
|  |  | KD_vs_KW | 1.235 | 0.004180 |  |
|  |  | KC_vs_KW | 1.182 | 0.001436 |  |
| Gene5174 | *papR* | KC_vs_KD | -1.736 | 0.0001920 | cell-cell signaling protein PapR |
|  |  | KD_vs_KW | -2.279 | 6.609 |  |
|  |  | KC_vs_KW | -3.998 | 2.315 |  |
| Gene1795 | *NheA* | KC_vs_KD | -2.952 | 8.341 | enterotoxin |
|  |  | KD_vs_KW | -2.740 | 2.028 |  |
|  |  | KC_vs_KW | -5.676 | 1.659 |  |
| Gene1796 | *NheB* | KC_vs_KD | -2.221 | 1.013 | enterotoxin |
|  |  | KD_vs_KW | -3.592 | 3.355 |  |
|  |  | KC_vs_KW | -5.795 | 3.622 |  |
| Gene1797 | *NheC* | KC_vs_KD | -3.538 | 1.813 | Hemolytic enterotoxin |
|  |  | KD_vs_KW | -0.9259 | 0.001429 |  |
|  |  | KC_vs_KW | -4.447 | 3.659 |  |
|  |  |  |  |  |  |

Note: KC indicated lag phase; KD indicated logarithmic phase; KW indicated stationary phase.

Table S8 Expression of *nheB* and *nheC* at lag, logarithmic, and stationary phases

| Gene name | Lag phase | Logarithmic phase | stationary phase |
| --- | --- | --- | --- |
| NheB | 23126.35±2763.37 | 5108.84±739.46 | 475.6±9.2 |
| NheC | 2653.58±315.37 | 235.09±33.8 | 138.81±2.72 |
| Rate | 8.7 | 21.7 | 3.4 |
